# THBS1 as a Key Regulator of Myoblasts: Validation of Its Inhibitory Roles in Skeletal Muscle Development

**DOI:** 10.3390/genes17060720

**Published:** 2026-06-21

**Authors:** Ji Qi, Xinlin Jin, Jing Wang, Yunzhou Wang, Wei Chen, Hongzhen Cao, Jingsen Huang, Hui Tang, Junfeng Chen, Baosong Xing, Yongqing Zeng

**Affiliations:** 1 Shandong Provincial Key Laboratory for Livestock Germplasm Innovation & Utilization, College of Animal Science and Technology, Shandong Agricultural University, Tai’an 271018, China; marica0817@163.com (J.Q.); xinlin_jin@163.com (X.J.); wchen@sdau.edu.cn (W.C.); hongzhencao@163.com (H.C.); hjs990606@163.com (J.H.); tanghui99@126.com (H.T.); 2 Henan Key Laboratory of Farm Animal Breeding and Nutritional Regulation, Henan Pig Breeding Engineering Research Centre, Institute of Animal Husbandry, Henan Academy of Agricultural Sciences, Zhengzhou 450002, China; wangjing@hnagri.org.cn (J.W.); afeng008@163.com (J.C.); 3 Department of Veterinary Medicine, Shandong Vocational Animal Science and Veterinary College, Weifang 261061, China; wyzwin01@163.com

**Keywords:** *THBS1*, myoblasts, TGF-β signaling pathway, skeletal muscle, cellular differentiation

## Abstract

Background: Thromboxane B2 Synthase 1 (THBS1), also known as TSP-1, is a multifunctional glycoprotein involved in various cellular processes that plays a crucial role in skeletal muscle development and repair. It acts as a key physiological activator of transforming growth factor-β1 (TGF-β1), both in vivo and in vitro. Methods: This study aimed to investigate the effects of THBS1 on myoblast proliferation and differentiation using the mouse skeletal muscle satellite cell line C2C12 and to reveal its regulatory relationship with the TGF-β signaling pathway through a series of cellular and molecular experiments. Results: Overexpression or knockout of *THBS1* in C2C12 cells regulates and upregulates the activity of the TGF-β signaling pathway, thereby inhibiting the proliferation and differentiation of C2C12 cells. Conclusions: These results provide a theoretical foundation for future research aimed at enhancing skeletal muscle quality in livestock and poultry and lay the groundwork for developing therapeutic strategies for skeletal muscle disorders.

## Simple Summary

Skeletal muscle plays a decisive role in the yield and quality of meat in mammals. It is regulated by a complex molecular network. Through transcriptome sequencing, we identified *THBS1* as a core differentially expressed gene; however, its specific regulatory functions in skeletal muscle remain unclear. In this study, we demonstrated that the THBS1 gene inhibits the proliferation and differentiation of C2C12 myoblasts. It was also found to enhance the activity of the TGF-β signaling pathway, thereby suppressing cell proliferation and differentiation. These findings provide significant theoretical support for the potential application of the *THBS1* gene as a molecular marker in regulating muscle growth and facilitating genetic improvements in livestock.

## 1. Introduction

Skeletal muscle is one of the tissues constituting the largest proportion of body weight in mammals and plays a decisive role in the meat yield and quality of livestock and poultry [1]. It also provides critical structural support and protection for vital organs [2]. The formation of skeletal muscle is strictly regulated by a complex molecular network that coordinates multiple key developmental stages [3]. Initially, mesenchymal stem cells differentiate into myoblasts, which proliferate through asymmetric division to generate proliferative myoblasts [4]. After a limited number of divisions, these myoblasts exit the cell cycle and enter a terminal differentiation program [5]. Differentiated cells fuse with each other via surface membrane proteins, forming multinucleated myotubes [6]. These myotubes undergo continuous fusion and cytoskeletal remodeling, ultimately maturing into contractile muscle fibers [7].

Thrombospondin 1(THBS1) has been implicated in various cancers, with elevated expression observed in breast cancer, thyroid cancer, lung adenocarcinoma, esophageal squamous cell carcinoma, melanoma, and oral squamous cell carcinoma [8]. Conversely, THBS1 expression is diminished in certain malignancies, including cervical cancer, glioma, and prostate cancer [9,10]. Moreover, THBS1 is known to act as an inhibitor of angiogenesis and tumorigenesis [11,12].

In aging muscle stem cells, changes in the expression and functional interaction between CD47 and its ligand THBS1 contribute to the gradual decline of muscle stem cell function [13]. Sarcopenia, characterized by the progressive loss of muscle mass and function, is not merely the result of skeletal muscle fiber atrophy, but is also associated with the functional exhaustion of muscle stem cells (MuSCs) [14]. Studies on the adhesion of THBS1 protein to skeletal muscle cells have identified multiple binding sites within the THBS1 molecule, including the amino-terminal globular domain, and heparin and sulfated guanosine binding sites within the type I domain [15]. THBS1 has been recognized as the primary physiological activator of transforming growth factor-β1 (TGF-β1) both in vivo and in vitro [16].

A study by Davy Vanhoutte et al. examined muscle phenotype using a skeletal muscle-specific THBS1 transgenic mouse model, generated via adenovirus-mediated gene overexpression and interference [17]. They found that THBS1 was upregulated under conditions of skeletal muscle atrophy, and its overexpression led to severe muscle atrophy. This phenomenon was driven by the activation of the TGF-β-Smad2/3 signaling pathway, which induces ATF4 expression, regulates the autophagy-lysosomal pathway (ALP), and activates the ubiquitin-proteasome system (UPS), ultimately promoting muscle atrophy. Specific inhibition of TGF-β receptor signaling or knockout of Smad2/3 in skeletal muscle significantly alleviated THBS1-induced muscle atrophy, indicating that the TGF-β-Smad2/3-ATF4 signaling axis plays a pivotal role in THBS1-induced muscle atrophy [18].

Currently, exploring the mechanisms regulating meat production performance and skeletal muscle quality is a key research focus in the livestock industry. In this study, THBS1 was identified as a core differentially expressed gene (DEG) based on the laboratory’s previously generated high-throughput whole-transcriptome sequencing data of Duroc pigs with divergent average daily gain (ADG), as well as single-nucleus transcriptome sequencing (snRNA-seq) data of Jiangquan black pigs. The effects of THBS1 on myoblast proliferation, cell cycle regulation, and differentiation were investigated using in vitro myoblast cell models. Furthermore, we examined the dynamic regulatory mechanisms of the TGF-β/Smad signaling pathway mediated by THBS1 in muscle cell development. The findings provide essential theoretical support for the potential application of THBS1 as a molecular marker in regulating muscle growth and facilitating genetic improvements in livestock.

## 2. Materials and Methods

### 2.1. Cell Culture and Differentiation

The mouse C2C12 cell line was purchased from Wuhan Puno Life Technology Co., Ltd. (Wuhan, China) Cells were cultured in complete medium within an incubator maintained at 5% CO_2_, 95% humidity, and 37 °C, with timely replacement of complete medium. When cell density reached 80–90%, cells were passaged at a 1:2 ratio or cryopreserved.

Induction of Differentiation: When cell density reached approximately 70%, differentiation was induced using differentiation induction medium (DMEM supplemented with 2% horse serum). The differentiation induction medium was replaced as needed.

### 2.2. Cell Transfection

The transfection reagents used were liposomal nucleic acid transfection reagent from Yisheng Biotechnology Co., Ltd. (Shanghai, China), and Lipofectamine TM2000 product from Invitrogen (Waltham, MA, USA). For cell transfection, we inoculated a certain number of cells into a six-well cell culture plate 20–24 h before transfection to achieve a cell culture density of 70%~95% during transfection. Transfection complexes were prepared according to the following ratios (per well): We diluted siRNA or overexpression vector with 100 μL Opti-MEM, and transfection reagent with 100 μL Opti-MEM (the corresponding amount of si RNA transfection reagent is 5 μL, and the ratio of overexpression plasmid to transfection reagent is 1:2). This was left to stand still for 5 min, before slowly adding the diluted transfection reagent dropwise into the diluted siRNA or overexpression plasmid solution. Then, we mixed it gently and incubated it at 15–25 °C for 20 min to form a transfection complex. The complete culture medium was discarded from the six-well plate and replaced with serum-free culture medium. Transfection complexes were added dropwise to each well, and the culture plate was shaken to gently mix the contents. Growth medium was changed after 4–6 h.

### 2.3. Construction of Overexpression Vectors and Extraction of Recombinant Plasmids

The mouse PCMV3-C-*THBS1* overexpression vector was provided by Beijing Yiqiao Shenzhou Technology Co., Ltd. (Beijing, China); the *THBS1* gene sequence accession number is NM_011580.3, and the plasmid sequence is shown in Appendix B.

The competent cells were removed from the −80 °C ultra-low temperature freezer and melted on ice. We then placed 100 μL of competent cells in a 1.5 mL sterile enzyme-free centrifuge tube. The constructed overexpression plasmid was added, gently mixed, and left to stand in an ice bath for 2 min. The centrifuge tube was exposed to heat shock in a 42 °C water bath for 30 s and then quickly transferred to an ice bath for 2 min, being careful not to shake the centrifuge tube. We added 200 μL of antibiotic-free LB medium, gently mixed it well, and incubated it on a shaker at 37 °C and 200 rpm for 1 h. We transferred the transformed competent cells onto antibiotic-resistant LB agar medium and spread the liquid evenly with a coating rod. We placed the plate at 37 °C until the liquid was diluted, inverted the plate, and incubated overnight. Then, we prepared 200 mL LB liquid culture medium, sterilized it with high-pressure steam, and let it cool. We added an appropriate amount of transformed bacterial solution and antibiotics, and incubated it overnight on a shaker at 37 °C and 200 rpm. We used the plasmid extraction kit from Tiangen Biochemical Technology Co., Ltd. (Beijing, China), to extract plasmids according to the instructions.

### 2.4. Synthetic siRNA and Negative Control

The mouse THBS1 gene sequence (accession number: NM_011580.4) was submitted to Shanghai Jima Biotechnology Co., Ltd. (Shanghai, China), which designed four pairs of siRNAs and one pair of negative controls (Negative Control siRNA, si-NC), with sequences shown in Table A1. Upon receipt of the siRNAs, they were briefly centrifuged to sediment at the tube bottom, followed by addition of 125 μL DEPC buffer for dissolution, achieving a final concentration of 20 μM. The diluted siRNAs were stored at –20 °C. The specific sequence is listed in Appendix A.1.

### 2.5. qRT-PCR

Total RNA was extracted using the Total RNA Extraction Kit provided by Sangon Biotech (Shanghai, China). The specific operation steps were as follows: 1 mL of lysis buffer was added to each well of a 6-well plate, and the mixture was pipetted repeatedly to fully lyse the cells and collect the lysate; 200 μL of chloroform was then added, followed by centrifugation at 4 °C, 12,000 rpm for 10 min. Next, 500 μL of the upper aqueous phase was carefully transferred to a new tube, mixed with 250 μL of absolute ethanol, and loaded into an RNA adsorption column for centrifugal filtration. The column was then washed with 500 μL of protein removal buffer and centrifuged at 12,000 rpm for 1 min to discard the flow-through, followed by an additional wash with 500 μL of wash buffer and centrifugation at 12,000 rpm for 1 min to remove residual buffer. The column was air-dried for 10 min to evaporate residual ethanol, and RNA was finally eluted with 30 μL of nuclease-free water via centrifugation at 12,000 rpm for 1 min.

cDNA synthesis was performed using the Evo M-MLV Reverse Transcriptase Premix Kit (Accurate Biology, Changsha, China) in a 20 μL reaction system following the manufacturer’s protocol.

Quantitative real-time PCR (qPCR) was carried out on a Roche LightCycler 96 Real-Time PCR System (Roche Diagnostics, Basel, Switzerland). The thermal cycling program was set as follows: an initial denaturation at 95 °C for 30 s, followed by 40 cycles of denaturation at 95 °C for 5 s and annealing/extension at 60 °C for 30 s. Each group includes three biological replicates and three technical replicates to minimize experimental error. The statistical significance of differences among groups was examined using a t-test or one-way ANOVA. The primer sequences used in this study are listed in Appendix A.2.

### 2.6. CCK-8 Cell Proliferation Detection

The CCK-8 cell proliferation detection kit was provided by Beyotime Biotechnology Co., Ltd. (Shanghai, China). First, 100 μL of cell suspension (per well) was inoculated into a 96-well plate and cultured in an incubator until the cell density reached 80% for transfection. We then added 10 μL of CCK-8 reagent to each well at 12 h, 24 h, 36 h, and 48 h and then incubated it in the dark for 2 h. We then measured the absorbance at 450 nm wavelength and calculated cell viability according to the following formula:

[experimental group-blank group]/[control group-blank group] × 100%.

### 2.7. EdU-488 Cell Proliferation Detection

The EdU-488 cell proliferation detection kit was provided by Beyotime Biotechnology Co., Ltd. (Shanghai, China) Crawling treatment was carried out by adding 100 μL of cell suspension onto the slides, incubating them in a culture box for 1 h, and then adding 2 mL of complete culture medium for transfection. We then added the prepared 2× working solution and incubated it for 2 h. We removed the culture solution and added 1 mL of fixative (4% paraformaldehyde) to each well. We then fixed it at room temperature for 15 min. We removed the fixative, added 1 mL PBS to each well, and washed three times. We gently shook and washed on a shaker for five minutes each time. Then, we added 1 mL of 0.3% Triton X-100 permeate to each well and incubated at room temperature for 10 min. We removed the permeate and washed each well three times with 1 mL of PBS for five minutes each time. We added 500 μL of pre-prepared Click reaction solution, gently shook the culture plate to mix it thoroughly, and incubated it at room temperature in the dark for 30 min. We removed the reaction solution and washed each well three times with 1 mL of PBS for five minutes each time. We added an appropriate amount of anti-fluorescence quenching sealing agent (including DAPI) on the slide, used tweezers to grind the edge of the slide, and gently removed it; the slide was placed upside down on the slide with sealing agent added, and the edge was sealed with nail polish. The cell proliferation was observed under a fluorescence inverted microscope.

### 2.8. Cell Cycle and Apoptosis Detection Using Flow Cytometry

Preparation of cycle cell samples: Cell culture medium was collected in a 1.5 mL centrifuge tube and washed twice with 1 mL PBS. We added 1 mL of trypsin, let it stand for 2 min, and then added the cell culture medium collected above. The cells were collected into a new 1.5 mL centrifuge tube and centrifuged at 1000 rpm for 5 min. We removed the supernatant, added 1 mL of PBS to resuspend the cells, and centrifuged at 1000 rpm for 5 min. Fixed cells were added to the prepared PI staining solution and stained; 500 μL of PI staining solution was added to each centrifuge tube, and the cells were slowly resuspended. Then, the cells were incubated in the dark at 37 °C for 30 min and tested on the machine.

Preparation of apoptotic cell samples: We sucked out the cell culture medium and placed it in a new 2 mL sterile enzyme-free centrifuge tube. We washed the cells once with PBS, added 1 mL of trypsin without EDTA to digest the cells, and let them stand at room temperature. We paid attention to digestion time and avoided digestion times that were too short or too long. We added the cell culture medium collected above, gently blew down the cells, and transferred them to a new centrifuge tube. They were centrifuged at 1000 rpm for 5 min. We discarded the supernatant, collected the cells, and resuspended the cells in PBS. The resuspended cells were centrifuged at 1000 rpm for 5 min. After discarding the supernatant, we added 195 μL of Annexin V-FITC binding solution and gently resuspended the cells. We added 5 μL of Annexin V-FITC and mixed gently, and then added 10 μL of PI staining solution and mixed gently. This was incubated at room temperature in the dark for 20 min, resuspended twice during the incubation process, and then placed in an ice bath. Machine testing was performed immediately.

### 2.9. Western Blot

C2C12 cells were lysed using RIPA lysis solution containing PMSF (Beyotime, Shanghai, China). The BCA protein assay kit (Beyotime, Shanghai, China) was used to quantify protein. The treated cells were cultured for the desired number of days, washed once with PBS, and then lysed with RIPA lysis solution containing PMSF and collected into 1.5 mL centrifuge tubes. Lysates were centrifuged at 12,000 rpm for 5 min at 4 °C, and the supernatant was collected. The supernatants were subjected to BCA protein analysis (Beyotime, Shanghai, China) for protein quantification. Protein samples were diluted to the same concentration with lysate, added to SDS-PAGE up-sampling buffer, then heated at 100 °C in a metal bath for 10 min, and stored at −20 °C. The 20 µg protein was loaded in each well and then separated via SDS-PAGE. Subsequently, proteins were transferred to a PVDF membrane. After blocking nonspecific sites with blocking solution, the PVDF membranes were sequentially incubated with primary antibodies overnight at 4 °C (the information on antibodies could be found in Appendix C.), washed three times with TBST, and then with secondary antibodies for 1 h. ECL luminescent solution was added to the PVDF membrane and incubated for 5 min away from any light; then, the protein bands were detected using a chemiluminescent imager.

### 2.10. Statistical Analysis

All data were analyzed using IBM SPSS Statistics 20 software. Each group includes three biological replicates and three technical replicates to minimize experimental error. The statistical significance of differences among groups was examined using a *t*-test or one-way ANOVA. Images were plotted using GraphPad Prism 5.0, with data for each group expressed as Mean ± SEM. Statistical significance is expressed as * *p* < 0.05; ** *p* < 0.01; *** *p* < 0.001.

## 3. Results

### 3.1. Expression of THBS1 in C2C12 Cell Differentiation Induction

**Figure 1:**
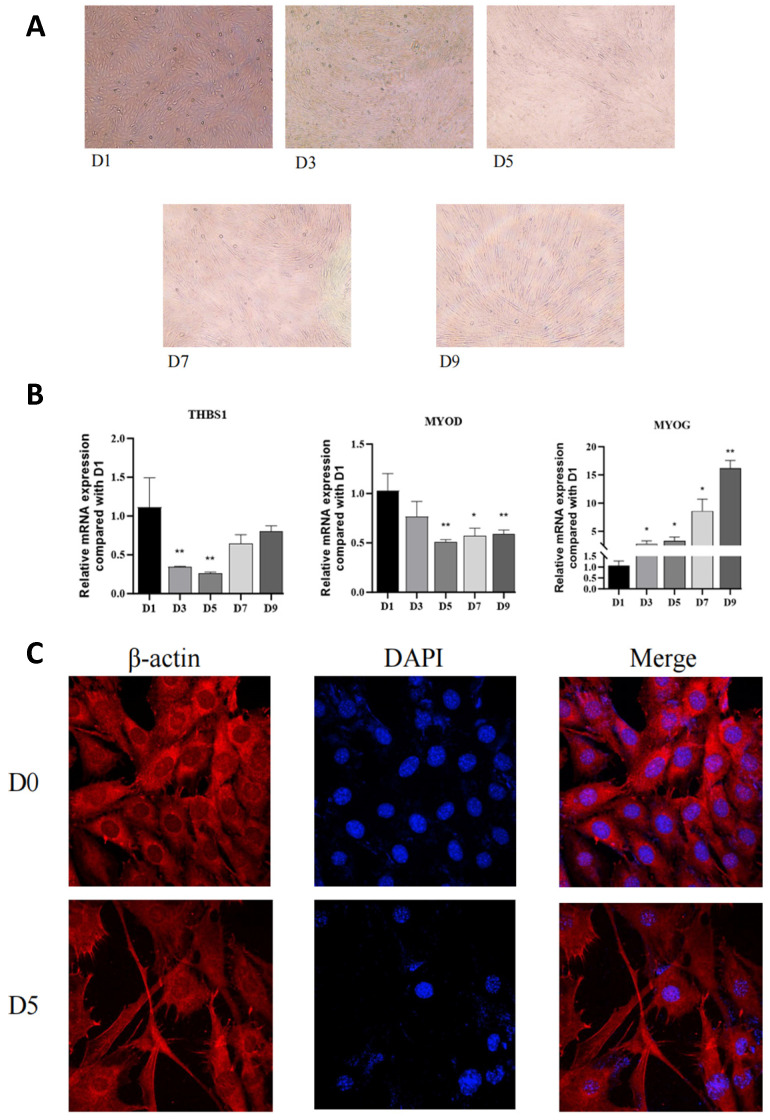
Expression of *THBS1* at different stages of C2C12 cell differentiation. (**A**) Myotube fusion state; (**B**) expression levels of *THBS1*, *MyoG*, and *MyoD* genes on each day of differentiation compared to D1; (**C**) analyzed by laser confocal microscopy, blue represents the nucleus (DAPI) and red represents the β-actin protein. Magnification is 630× (* *p* < 0.05,** *p* < 0.01).

Differentiation induction medium was used to induce differentiation of cells. Their differentiation status was observed under a microscope at D1, D3, D5, D7, and D9 days, as shown in Figure 1A. At D0 and D5 days, fluorescence staining of the cytoskeleton (β-actin) was performed. The results are shown in Figure 1C and indicate a good state of cell differentiation. The expression of *THBS1*, *MyoG*, and *MyoD* was detected in differentiated cells on days D1, D3, D5, D7, and D9, as shown in Figure 1B. The expression of the *THBS1* gene showed a significant downward trend followed by an upward trend. The expression level of *MyoG* gene showed a significant increase with the increase in differentiation days. The *MyoD* gene showed a trend of first decreasing and then increasing, and the trend was significant at D5.

### 3.2. Subcellular Localization of THBS1

**Figure 2:**
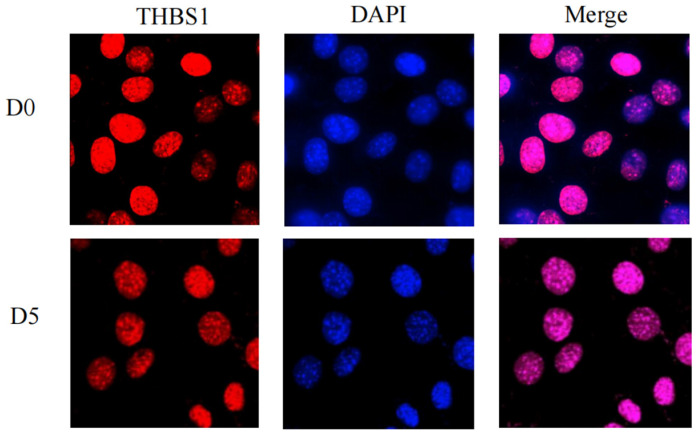
CelluLar sub-localization assay of THBS1 protein, analyzed using laser confocal microscopy. Blue represents nuclei (DAPI), red represents THBS1 protein. Magnification is 630×.

Cell differentiation was induced using differentiation induction medium and culture until day 5. Immunofluorescence staining was employed on D0 and D5 to analyze the subcellular localization of THBS1 in myoblasts, and the cells were photographed under a confocal laser microscope. As shown in Figure 2, THBS1 is expressed in the nucleus on both D0 and D5.

### 3.3. The Effect of THBS1 on Apoptosis of C2C12 Cells

**Figure 3:**
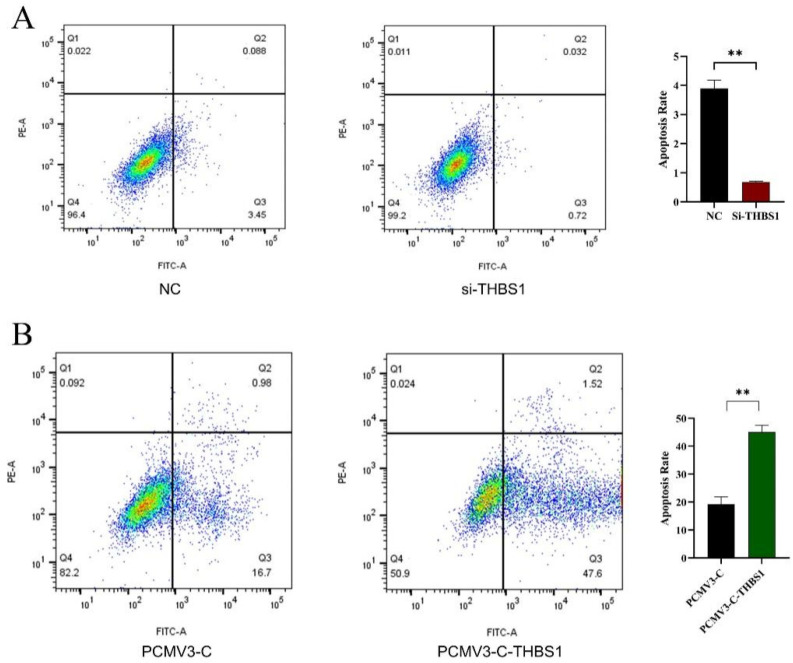
Effect of *THBS1* on apoptosis of C2C12 cells determined by flow cytometry. (**A**) Detection of apoptosis after interference with *THBS1* and apoptosis rate; (**B**) detection of apoptosis after overexpression of *THBS1* and apoptosis rate (** *p* < 0.01).

The interfering fragment and overexpression vector of the *THBS1* gene were transfected into C2C12 cells, and apoptosis was detected using an apoptosis detection kit and flow cytometry. As shown in Figure 3, overexpression of the *THBS1* gene (Figure 3A) significantly increased the number of apoptotic cells (*p* < 0.01), while interference with the *THBS1* gene (Figure 3B) significantly reduced the number of apoptotic cells (*p* < 0.01). This demonstrates that overexpression of the *THBS1* gene promotes apoptosis in C2C12 cells.

### 3.4. Effect of THBS1 on the Proliferation of C2C12 Cells

#### 3.4.1. Flow Cytometry Detection of C2C12 Cell Cycle

**Figure 4:**
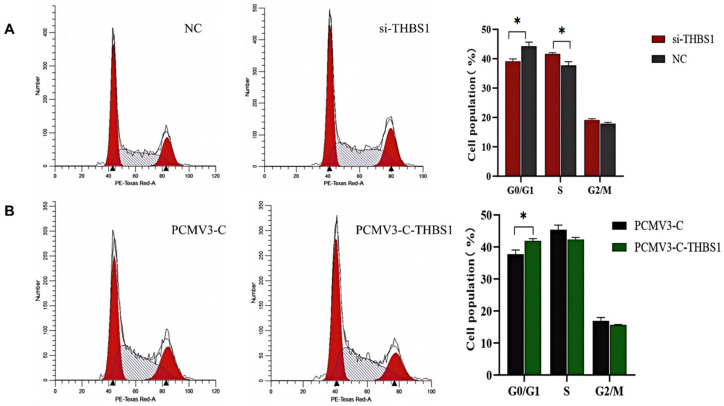
Effects of *THBS1* on C2C12 cell cycle as determined by flow cytometry. (**A**) Cell cycle assay after interference with *THBS1* and the number of cells in each period of the cell cycle; (**B**) cell cycle assay after overexpression of *THBS1* and the number of cells in each period of the cell cycle (* *p* < 0.05).

The interfering fragment and overexpression vector of *THBS1* gene were transfected into C2C12 cells, and cell cycle and apoptosis were detected using a cell cycle and apoptosis detection kit and a flow cytometer. As shown in Figure 4, after overexpression of the *THBS1* gene (Figure 4A), the number of cells in the G0/G1 phase significantly increased (*p* < 0.05), while the number of cells in the S phase decreased, indicating that overexpression of the *THBS1* gene can cause cells to stagnate in the G0/G1 phase and inhibit DNA synthesis. After interfering with the *THBS1* gene (Figure 4B), there was a significant decrease in G0/G1 phase cells and a significant increase in S phase cells (*p* < 0.05). Interference with the *THBS1* gene can prolong the S phase of cells, promote DNA synthesis, and thus promote cell proliferation. These results indicate that the *THBS1* gene has an inhibitory effect on cell proliferation.

#### 3.4.2. The Effect of *THBS1* on the C2C12 Cell Proliferation Activity

**Figure 5:**
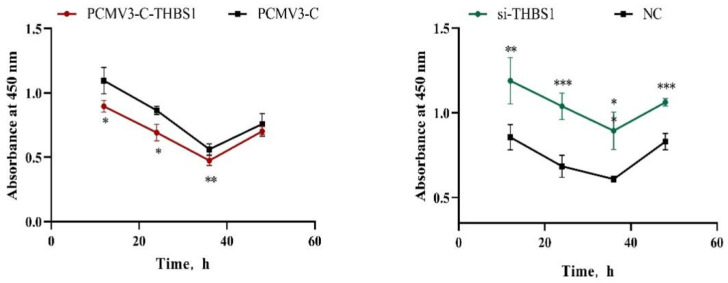
Effects of *THBS1* on C2C12 cell cycle was determined by CCK-8 (* *p* < 0.05, ** *p* < 0.01, *** *p* < 0.001).

The interference fragment and overexpression vector of the *THBS1* gene were transfected into C2C12 cells, and cell proliferation activity was detected using CCK-8 reagent at 12 h, 24 h, 36 h, and 48 h. The OD values measured at a wavelength of 450 nm are shown in Figure 5. Compared with the control group, overexpression of *THBS1* significantly reduced cell activity at 12 and 24 h (*p* < 0.05) and significantly decreased cell activity at 36 h (*p* < 0.01). Interference with *THBS1* significantly increased cell viability at 12, 24, and 48 h (*p* < 0.01) and at 36 h (*p* < 0.05), indicating that the *THBS1* gene can inhibit C2C12 cell viability.

#### 3.4.3. EDU Detection of C2C12 Cell Proliferation Ability

**Figure 6:**
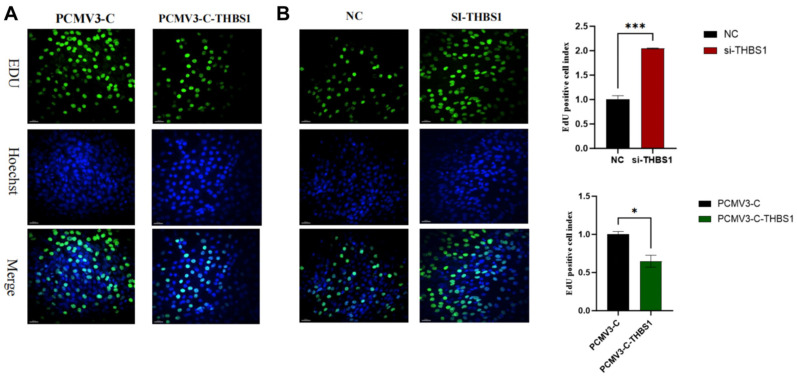
Effect of EdU detection of *THBS1* on cell proliferation. Blue represents cell nuclei, and EdU (green) fluorescence represents cell proliferation. (**A**) EdU cell assay after overexpression of *THBS1* gene; (**B**) EdU cell assay after interfering with *THBS1* gene. Magnification is 400× (* *p* < 0.05, *** *p* < 0.001).

The interference fragment and overexpression vector of the *THBS1* gene were transfected into C2C12 cells. The cells were stained with EdU, and fluorescence was detected under a fluorescence microscope. As shown in Figure 6, after interfering with the *THBS1* gene, the positive rate of EdU cells (green fluorescence) significantly increased. Overexpression of the *THBS1* gene significantly reduced the positivity rate of EdU cells.

#### 3.4.4. The Effect of *THBS1* on Cell Proliferation and Differentiation

**Figure 7:**
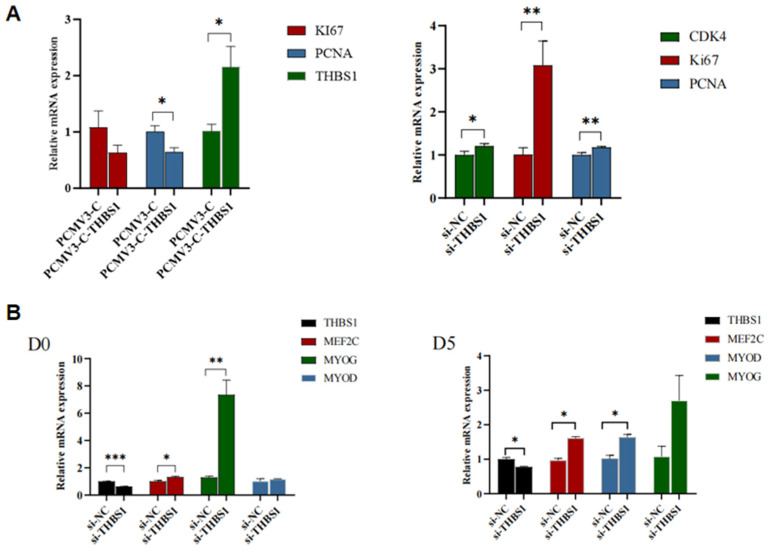
Effects of *THBS1* on proliferation marker genes and differentiation marker genes in C2C12 cells detected by qRT-PCR. (**A**) Proliferation marker gene assay after overexpression of *THBS1* gene and interference with *THBS1* gene (* *p* < 0.05, ** *p* < 0.01); (**B**) Assay for differentiation marker genes at D0 and D5 days after interference with the *THBS1* gene (* *p* < 0.05, ** *p* < 0.01, *** *p* < 0.001).

The interference fragment and overexpression vector of *THBS1* gene were transfected into C2C12 cells at corresponding concentrations. The cells were cultured for 24–48 h and then collected to detect the expression of proliferation marker genes *CDK4*, *Ki67*, and *PCNA*. As shown in Figure 7A, overexpression of *THBS1* significantly reduced the expression of proliferation marker *PCNA* (*p* < 0.05) and *Ki67*. After interfering with *THBS1*, the expression levels of proliferation marker genes *PCNA* and *Ki67* in the si-*THBS1* group were significantly increased (*p* < 0.01), as was the expression level of *CDK4* (*p* < 0.05). This indicates that the *THBS1* gene has the ability to inhibit cell proliferation. At D5, we used qRT-PCR technology to detect the expression of differentiation marker genes *MyoD*, *MyoG*, and *MEF2C*. The results are shown in Figure 7B. At D5 days, compared with the si NC group, the expression levels of *MEF2C* and *MyoD* in the si-*THBS1* group significantly increased (*p* < 0.05), and the expression level of *MyoG* showed a non-significant increase.

### 3.5. WB Detection of Cell Differentiation

**Figure 8:**
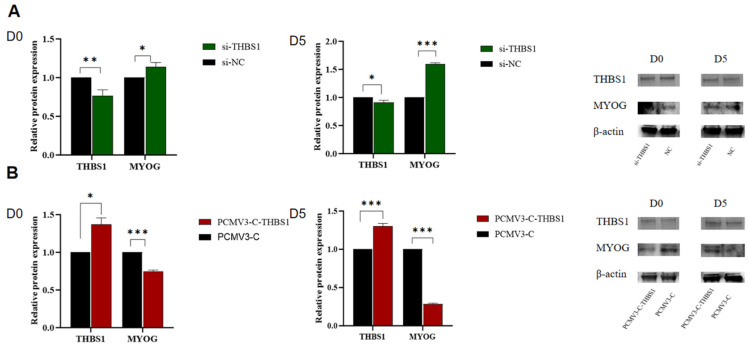
(**A**) Effects of interfering *THBS1* on differentiation of C2C12 cell differentiation; (**B**) effect of overexpression of *THBS1* on differentiation of C2C12 cell differentiation (* *p* < 0.05, ** *p* < 0.01, *** *p* < 0.001).

Western blot technology was used to detect the expression levels of differentiation marker protein MyoG. As shown in Figure 8A, compared to the si-NC group, the si-*THBS1* group exhibited significantly elevated MyoG protein levels at D0 (*p* < 0.05) and markedly elevated MyoG protein levels at D5 (*p* < 0.01). As shown in Figure 8B, compared to the PCMV3-C group, the PCMV3-C-*THBS1* group exhibited a significantly reduced MyoG protein level on day D0 (*p* < 0.01) and a markedly decreased MyoG protein level on day D5 (*p* < 0.01). These results indicate that overexpression of the *THBS1* gene has an inhibitory effect on cell differentiation. The analysis of overexpression and interference results indicates that the *THBS1* gene has the ability to inhibit cell differentiation.

### 3.6. The Effect of TGF-β Signaling Pathway on the Proliferation and Differentiation of C2C12 Cells

**Figure 9:**
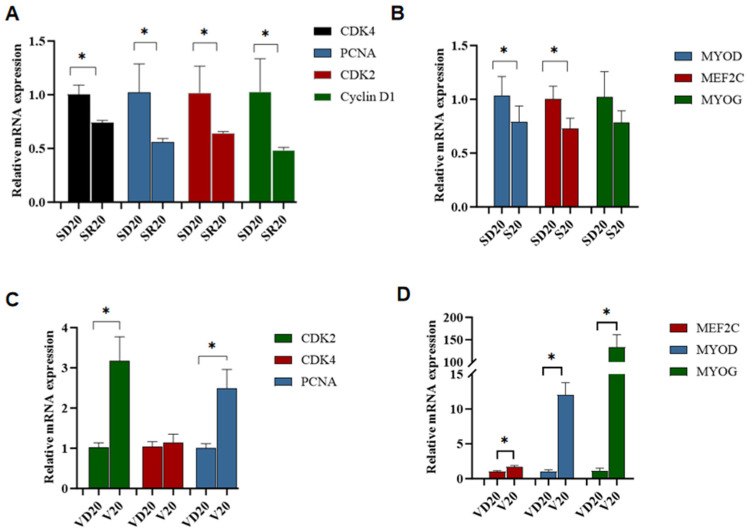
Real-time quantitative fluorescence PCR (qRT-PCR) analysis of the regulatory effects of the TGF-β signaling pathway on proliferation and expression of differentiation marker genes in C2C12 cells. (**A**) Addition of the SR1-01138 signaling pathway activator and analysis of the expression levels of genes associated with pathway proliferation markers. (**B**) Addition of the SR1-01138 signaling pathway activator and analysis of the expression levels of genes associated with pathway differentiation markers. (**C**) Addition of a Viatertide signaling pathway inhibitor alters the expression levels of genes associated with pathway proliferation. (**D**) Addition of a Viatertide signaling pathway inhibitor alters the expression levels of genes associated with pathway differentiation (* *p* < 0.05).

In this study, cells were treated with the TGF-β signaling pathway activator SR1-01138 and the inhibitor Viatertide during cell culture and induced differentiation. Four experimental groups were set up: the group treated with 20 μmol/L SR1-01138 (designated SR20), the group treated with 20 μmol/L Viatertide (designated V20), and the negative control group supplemented with equal volume of DMSO. Among these, the vehicle control corresponding to the SR1-01138 treatment was designated D20, and the vehicle control corresponding to the Viatertide treatment was designated VD20. We collected cells and detected the expression of differentiation marker genes for proliferation using qRT-PCR technology. As shown in Figure 9A, the activation of the TGF-β signaling pathway significantly reduced the expression levels of proliferation marker genes (*CDK4*, *PCNA*, *CDK2*, and *Cyclin D1*) (*p* < 0.05). As shown in Figure 9B, inhibition of the TGF-β signaling pathway significantly reduced the expression levels of proliferation marker genes (*PCNA* and *CDK2*) (*p* < 0.05), indicating that the TGF-β signaling pathway has the ability to inhibit C2C12 cell proliferation.

As shown in Figure 9C, activation of the TGF-β signaling pathway significantly reduced the expression levels of differentiation marker genes (*MyoD* and *MEF2C*) (*p* < 0.05). As shown in Figure 9D, inhibition of the TGF-β signaling pathway significantly reduced the expression levels of differentiation marker genes *(MyoG*, *MyoD*, and *MEF2C*) (*p* < 0.05), indicating that the TGF-β signaling pathway has the ability to inhibit C2C12 cell proliferation and C2C12 cell differentiation.

### 3.7. The Effect of THBS1 Gene on TGF-β Signaling Pathway

**Figure 10:**
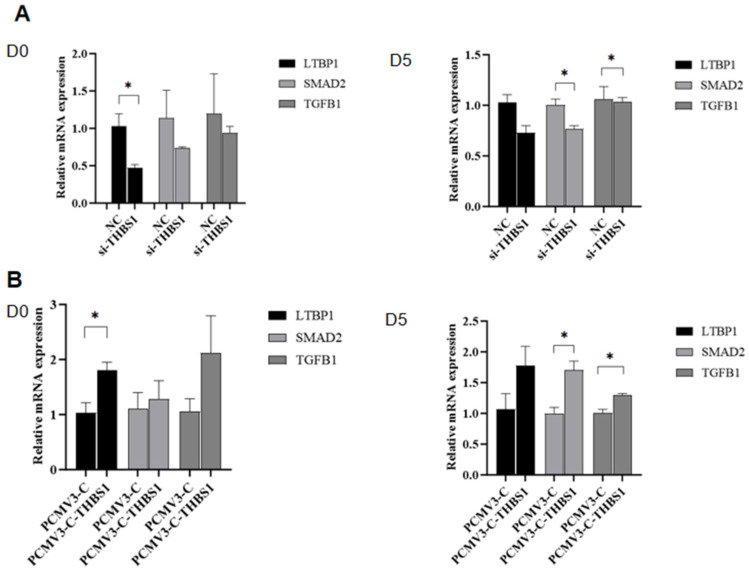
Detection of genes related to signaling pathways detected by qRT-PCR. (**A**) Detection of pathway-associated genes at D0 and D5 after interference with the *THBS1* gene; (**B**) detection of pathway-related genes at D0 and D5 after overexpression of *THBS1* gene (* *p* < 0.05).

We transfected the interfering fragment of the *THBS1* gene into C2C12 cells at the corresponding concentrations, induced cell differentiation, and treated the cells with activators and inhibitors of signaling pathways. Cells were cultivated to D5, and collected from D0 and D5. qRT-PCR technology was used to detect the expression levels of pathway-related factors (TGFB1, SMAD2, LTBP1). As shown in Figure 10A, the expression level of LTBP1 significantly decreased (*p* < 0.05) and the expression levels of TGFB1 and SMAD2 decreased at D0. At D5, the expression levels of TGFB1 and SMAD2 were significantly reduced (*p* < 0.05), and the expression level of LTBP1 was non-significantly reduced.

The same experiment was conducted using the overexpression vector of the *THBS1* gene. As shown in Figure 10B, the expression level of *LTBP1* significantly increased at D0 (*p* < 0.05), while the expression levels of *TGFB1* and *SMAD2* increased. At D5, the expression levels of *TGFB1* and *SMAD2* significantly increased (*p* < 0.05), and the expression level of *LTBP1* increased.

### 3.8. Interference with THBS1 and Simultaneous Activation of TGF-β Signaling Pathway

**Figure 11:**
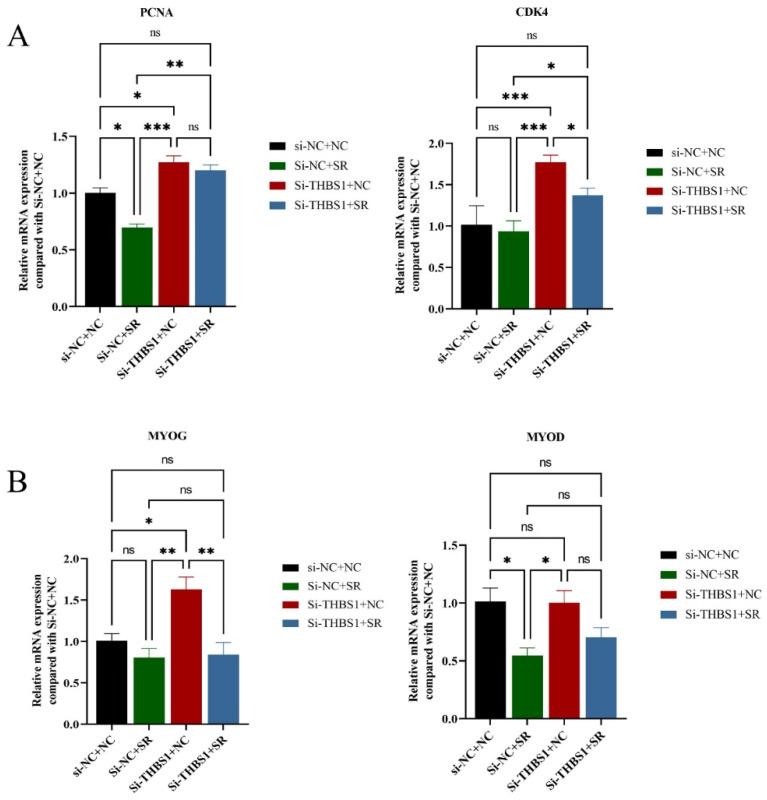
Detection of proliferation and differentiation marker genes by qRT-PCR. (**A**) Expression levels of PCNA and CDK4 after interfering with *THBS1* while activating the TGF-β signaling pathway; (**B**) Expression levels of MyoG and MyoD after interfering with *THBS1* while activating the TGF-β signaling pathway (* *p* < 0.05, ** *p* < 0.01, *** *p* < 0.001, “ns” means *p* > 0.05).

To verify whether the *THBS1* gene affects cell differentiation through the TGF-β signaling pathway, we first introduced interfering fragments into the cells and then treated them with signaling pathway activators (SR). We collected cell samples at D0 and D5 of differentiation and measured the expression levels of proliferation marker genes *PCNA* and *CDK4* at D0 using real-time quantitative PCR (qRT-PCR). We also detected the expression levels of differentiation marker genes *MyoD* and *MyoG* at D5 of differentiation. As shown in Figure 11A, at D0, compared with the si-*THBS1* + NC group and the si-NC + NC group, the expression of *PCNA* was significantly increased (*p* < 0.05), and the expression of *CDK4* was extremely significantly increased (*p* < 0.001). Compared with the si-NC + NC group, the si-NC + SR group showed a significant decrease in *PCNA* expression (*p* < 0.05) and a decrease in *CDK4* expression. The expression levels of *PCNA* and *CDK4* were significantly increased in the si-*THBS1* + NC group compared to the si-NC + SR group (*p* < 0.001). There was no significant difference in *PCNA* expression between the si-*THBS1* + SR group and si-*THBS1* + NC group, while the expression of *CDK4* was significantly reduced (*p* < 0.05). This indicates that interference with *THBS1* can, to some extent, rescue the proliferation inhibition caused by activators.

Figure 11B shows that at D5, compared with the si-*THBS1* + NC group and the si-NC + NC group, the expression level of *MyoG* significantly increased (*p* < 0.05) and the expression level of *MyoD* increased. Compared with the si-NC + NC group, the si NC + SR group showed a significant decrease in *MyoD* expression (*p* < 0.05) and a decrease in *MyoG* expression. Compared with the si-NC + SR group, the si-*THBS1* + NC group showed a significant increase in *MyoG* expression (*p* < 0.01) and *MyoD* expression (*p* < 0.05). Compared with si-*THBS1* + NC, the expression levels of *MyoG* and *MyoD* in the si-*THBS1* + SR group were significantly reduced (*p* < 0.01). The results indicate that interference with *THBS1* may, to some extent, salvage the differentiation inhibition caused by activators.

## 4. Discussion

Skeletal muscle development is a complex process governed by a precise network of regulatory genes and signaling pathways [19]. Our study identifies THBS1 as a novel negative regulator of MyoGenesis, exerting its effects primarily by modulating the transforming growth factor-beta (TGF-β) signaling pathway in C2C12 myoblasts. The findings demonstrate that *THBS1* inhibits both proliferation and differentiation, positioning it as a potential key node in the regulatory circuitry controlling muscle mass. In a mouse model, knockdown of *THBS1* weakened the *THBS1*-TGF-β-Smad2/3-ATF4 signaling pathway and reduced skeletal muscle atrophy induced by denervation and nutritional stress. This study indicates that THBS1 is a necessary endogenous mediator of muscle atrophy [20].

A series of gain- and loss-of-function experiments consistently established the inhibitory role of *THBS1*. This identified *THBS1* as a significantly downregulated gene associated with myogenesis and energy metabolism in neonatal Hanwoo calves under maternal overnutrition conditions [21]. Studies have demonstrated that in C2C12 myoblasts, *THBS1* expression levels gradually decline from day 2 to day 6 during the transition from adhesion to differentiation, consistent with the findings of this study. However, our analysis revealed a rebound in *THBS1* expression on day 9 of differentiation, which may be associated with the completion of myoblast fusion [22]. Overexpression of *THBS1* significantly suppressed myoblast proliferation, induced G0/G1 cell cycle arrest, and promoted apoptosis. This is consistent with findings in HCC-related research, where knockdown of miR-222-3p can directly target the overexpression of *THBS1*, thereby inhibiting the progression of HCC in vitro and suppressing cell proliferation [23]. Conversely, *THBS1* knockdown enhanced proliferation, facilitated S-phase entry, and reduced apoptosis. These phenotypic changes were corroborated by corresponding alterations in the expression of key proliferation markers (*PCNA*, *Ki67*, *CDK4*). Furthermore, *THBS1* knockdown led to the upregulation of critical MyoGenic regulatory factors *(MyoD*, *MyoG*, *MEF2C*) and their protein products, whereas overexpression suppressed them. This collective evidence strongly suggests that *THBS1* acts as a brake on both the expansion and the differentiation of the myoblast pool. The TGF-β pathway is a well-documented inhibitor of MyoGenesis [24,25,26]. Our data confirm its role in C2C12 cells, as pathway activation was suppressed, while its inhibition enhanced, the expression of both proliferation and differentiation markers. Previous studies have shown that THBS1 regulates the proliferation and apoptosis of MDCK cells through the TGF-β/Smad signaling pathway [27]. Given that *THBS1* is a known physiological activator of latent TGF-β [28], we investigated the functional interplay between them. The observation that *THBS1* knockdown could partially rescue the proliferation inhibition caused by TGF-β pathway activation provides compelling evidence that *THBS1* functions, at least in part, through this pathway. The more pronounced rescue effect on proliferation markers compared to differentiation markers suggests that the *THBS1*-TGF-β axis exerts a stronger influence on controlling cell cycle progression, while its impact on terminal differentiation may involve additional, parallel mechanisms or be secondary to the proliferation arrest.

In conclusion, our study delineates a model wherein *THBS1* serves as an upstream modulator that fine-tunes the TGF-β signaling pathway to restrain myoblast proliferation and differentiation. This not only expands our understanding of the molecular mechanisms controlling MyoGenesis but also highlights *THBS1* as a potential target for strategies aimed at enhancing muscle growth in livestock or countering muscle-wasting conditions. Future work should focus on validating these findings in vivo and elucidating the potential TGF-β-independent mechanisms through which *THBS1* may influence muscle cell fate.

## 5. Limitations of the Study

While this study has demonstrated that *THBS1* inhibits the proliferation and differentiation of C2C12 myogenic cells by regulating the TGF-β signaling pathway, several limitations remain that require further investigation. To date, the regulatory function of *THBS1* has only been validated in vitro cell models; no primary pig skeletal muscle cell model has been established, and future experiments will employ such cells for verification. Although this study confirms that *THBS1* exerts its effects via modulation of the TGF-β pathway, the upstream-downstream regulatory relationship between the two pathways remains unclear, and the mechanistic evidence for TGF-β pathway activation is incomplete. Subsequent studies should consider additional validation through protein phosphorylation assays.

## 6. Conclusions

The *THBS1* gene plays a crucial regulatory role in the proliferation and differentiation of C2C12 myoblasts. Overexpression of the *THBS1* gene can inhibit the expression of genes related to myoblast proliferation and differentiation, while interference with *THBS1* has the opposite effect. *THBS1* suppresses the proliferation and differentiation processes of myoblasts by affecting the TGF-β signaling pathway. A series of experiments revealed that overexpression of the *THBS1* gene promotes the activity of the TGF-β signaling pathway, which exerts an inhibitory effect on the proliferation and differentiation of myoblasts. Additionally, interference with the *THBS1* gene can reverse the inhibitory effects on proliferation and differentiation caused by the activation of the TGF-β signaling pathway. This study used an in vitro myoblast model to clarify the role of thrombospondin-1 (*THBS1*) in the molecular regulatory network of muscle growth and development, providing novel molecular targets for deciphering the genetic regulatory mechanisms of muscle. Future work will further refine the *THBS1* regulatory network by characterizing its upstream transcriptional regulatory elements, interacting proteins, and cross-talk with other key pathways of muscle development.

## Figures and Tables

**Figure A1:**
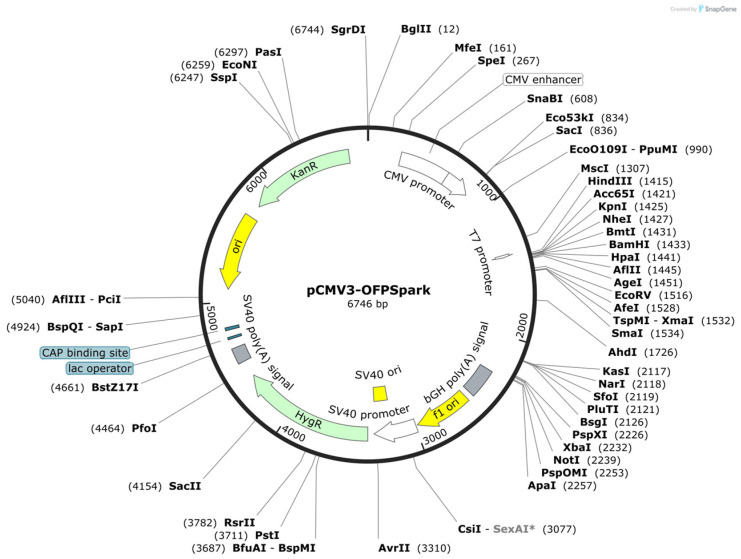
Mapping of mouse *THBS1* gene overexpression vectors.

**Figure A2:**
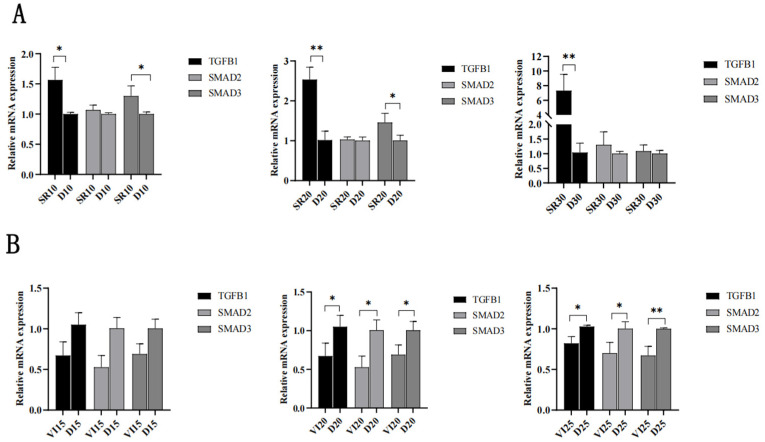
Detection of genes related to signaling pathways were detected by RT-qPCR (**A**) Expression of pathway-related genes at different concentrations of signaling pathway activators (**B**) Expression of pathway-related genes at different concentrations of signaling pathway inhibitors (* *p* < 0.05, ** *p* < 0.01).

## Data Availability

All the original data involved in this experiment can be obtained from the author.

## Appendix A

### Appendix A.1

The mouse *THBS1* gene sequence (accession number: NM_011580.4) was submitted to Shanghai Jima Biotechnology Co., Ltd., which designed four pairs of siRNAs and one pair of negative controls (Negative Control siRNA, si-NC), with sequences shown in Table A1. Upon receipt of the siRNAs, they were briefly centrifuged to sediment at the tube bottom, followed by addition of 125 μL DEPC buffer for dissolution, achieving a final concentration of 20 μM. The diluted siRNAs were stored at –20 °C.

**Table A1:** Sequence information of siRNA interference.

Gene	Forward (5′–3′)	Reverse (5′–3′)
*THBS1*-Mus-2667	GCCAGUACGUUUACAACGUTT	ACGUUGUAAACGUACUGGCTT
*THBS1*-Mus-3044	GUGCCAGAUAUUGAUGACATT	UGUCAUCAAUAUCUGGCACTT
*THBS1*-Mus-3582	CCGGUUAUAUCAGAGUGGUTT	ACCACUCUGAUAUAACCGGTT
*THBS1*-Mus-2875	GGCCGACCAUGAUAAAGAUTT	AUCUUUAUCAUGGUCGGCCTT
Si-NC	UUCUCCGAACGUGUCACGUTT	ACGUGACACGUUCGGAGAATT

### Appendix A.2

Primer sequences were designed using Primer 5.0 software; the fluorescent quantitative primer sequence information is shown in Table A2.

**Table A2:** The primer sequences of RT-qPCR.

Gene	Forward (5′–3′)	Reverse (5′–3′)
Mus-*THBS1*	CCTGGACTTGCTGTAGGTTAT	AGGACTGGGTGACTTGTTTC
Mus-*MyoD*	TCCGCTACATCGAAGGTCTG	GTCCAGGTGCGTAGAAGGC
Mus-*MyoG*	CGGGCTATGAGCGGACTGAG	CACAGACTTCCTCTTACACACCTTAC
Mus-*Mef2c*	GCAGCAGCAGCACCTACATAAC	TGAGTAGAAGGCAGGGAGAGATTTG
Mus-*CDK4*	GATGTCTGTGCTACTTCCCGAACTG	TGTCCTCAGGTCCTGGTCTATATGC
Mus-*CDK2*	ACTACTCTACAGCCGTGGACATC	CAATCTCAGAATCTCCAGGGAACAG
Mus-*Cyclin*	GAGGCGGATGAGAACAAGCAGAC	GGAGGGTGGGTTGGAAATGAACTTC
Mus-*PCNA*	AGAGGAGGAAGCAGTTACCATAGAG	ACATACTGAGTGTGACTGTAGGAGAG
Mus-*LTBP1*	ATCTTCCCTTGCCCCGTCCAG	GTTCTCGCCACCAGTGTCGTAAG
Mus-*TGFB1*	GCAACAATTCCTGGCGTTACCTTG	AGCCCTGTATTCCGTCTCCTTGG
Mus-*SMAD2*	GAGACCTTCCATGCGTCACAGC	GGAGAGCAAGCCTAAGCAGAACC
Mus-*SMAD3*	TCGTCCATCCTGCCCTTCACC	ACTTCTCCTCCTGCCCGTTCTG
Mus-*β-actin*	GTGACGTTGACATCCGTAAAGA	GCCGAACTCATCGTACTCC

### Appendix A.3

With three technical replicates per sample, experiments were conducted using the reaction systems specified in Table A3 and the procedures outlined in Table A4, performed on ice to minimize operational errors. Prior to primer use, preliminary experiments were conducted to establish relative standard curves, determine appropriate cDNA template concentrations, and verify primer specificity. The relative expression levels of each gene were calculated using the 2^−ΔΔCt^ method.

**Table A3:** Reaction system of real-time PCR.

Component Name	Volume (μL)
2×SYBR^®^ Green Pro Taq HS Premix	10.0
Primer F (10 uM)	0.4
Primer R (10 uM)	0.4
Template cDNA	2.0
RNase free water	7.2
Total	20.0

**Table A4:** Reaction program of real-time PCR.

Cyclic Step	Temperature (°C)	Time (s)	Cycle Number
Step 1	95	30	1
Step 2	95	5	40
	60	30	
Step 3		Dissociation Stage	

## Appendix B

The mouse PCMV3-C-*THBS1* overexpression vector was provided by Beijing Yiqiao Shenzhou Technology Co., Ltd. (Beijing, China); the *THBS1* gene sequence accession number is NM_011580.3, and the plasmid sequence is shown in Figure A1.

## Appendix C

### Appendix C.1

Detailed information about the antibodies used in this study is provided in Table A5.

**Table A5:** Antibody.

Antibody	Company of Production	Catalog Number	Dilute Strength
GAPDH	Proteintech	Cat No. 10494-1-AP	1:1000
THBS1	Proteintech	Cat No. 67241-1-Ig	1:1000
MyoG	Proteintech	Cat No. 26762-1-AP	1:1000

### Appendix C.2

To investigate the effects of the TGF-β signaling pathway on C2C12 cells, this study employed the TGF-β signaling pathway activator SR1-01138 and inhibitor Viatertide. The activator was added to C2C12 cells at concentrations of 10 μmol/L, 20 μmol/L, and 30 μmol/L during culture, while the inhibitor was added at concentrations of 15 μmol/L, 20 μmol/L, and 25 μmol/L. Expression levels of pathway-related factors (*TGFB1*, *SMAD2*, and *SMAD3*) were subsequently measured using RT-qPCR, as shown in Figure A2. The concentration of 20 μmol/L was ultimately selected for subsequent experiments.
